# Ampullary and Pancreatic Neuroendocrine Tumors: A Series of Cases and Review of the Literature

**DOI:** 10.7759/cureus.21657

**Published:** 2022-01-27

**Authors:** Venkata Vinod Kumar Matli, Gregory Wellman, Sathya Jaganmohan, Kirtan Koticha

**Affiliations:** 1 Internal Medicine, Christus Highland Medical Center, Shreveport, USA; 2 Gastrointestinal and Liver Pathology, Christus Highland Medical Center, Shreveport, USA; 3 Gastroenterology and Hepatology, Christus Highland Medical Center, Shreveport, USA; 4 Hematology and Oncology, Christus Highland Medical Center, Shreveport, USA

**Keywords:** pancreatic malignancy, gastrointestinal carcinoid tumor, pancreatic neuroendocrine tumors, pancreatic neuroendocrine tumor, ampullary carcinoids, ampullary neuroendocrine tumors, ampullary mass

## Abstract

The ampulla of Vater is a unique, highly vascularized pouch. Its anatomic transition is halfway along the second part of the duodenum from the foregut to the midgut. According to the World Health Organization’s (WHO) latest nomenclature, carcinoid tumors are now called neuroendocrine tumors (NETs). Knowledge of NETs is important because of their rarity, reclassification, prognosis, and management. NETs involving the ampulla of Vater are extremely rare, constituting <0.05% of gastrointestinal NETs, and involving the pancreas are rare to our knowledge. There are only a few reports of ampullary NETs.

We report two rare NET cases involving the ampulla and pancreas and review the relevant literature. A 71-year-old patient with neurofibromatosis and multiple comorbidities presented with chronic intermittent abdominal pain. Abdominal imaging studies showed a suspicious mass at the level of the ampulla. Endoscopic retrograde cholangiopancreatography and endoscopic ultrasound revealed bulging papillae. Histopathology of the biopsied mass revealed a low-grade NET. Given his multiple comorbidities, the patient was scheduled for endoscopic resection. Our second patient was an 83-year-old lady presenting with nausea and vomiting. Abdominal imaging studies revealed a lobular mass over the body of the pancreas. Histopathological examination of fine-needle aspiration of the mass confirmed a well-differentiated low-grade NET. Octreoscan and dotatate scans showed pancreatic, multiple hepatic and metastatic lesions in the left lung and left shoulder. The patient is currently stable after completing peptide receptor radioligand therapy at a tertiary oncology center. Because of the patients’ comorbidities and staging, their management has taken different approaches.

More data and more research are needed for accurate assessment of prognosis; however, a review of the latest literature recommends Whipple resection with lymphadenectomy for all ampullary NETs provided patients can tolerate the procedure. Endoscopic resection or surgical ampullary resection should be performed on contraindicated patients. The majority of ampullary and pancreatic NETs would have metastasized by the time patients sought treatment. Because of their rarity and ill-defined and highly variable presentation, NET diagnosis is always delayed and sometimes incidental; therefore, we emphasize the importance of early diagnosis and management to reduce mortality and morbidity.

## Introduction

The ampulla of Vater, also known as the hepatopancreatic duct, was named and first published in 1720 by the German anatomist Abraham Vater [1,2]. The ampulla is unique in its anatomic location and histophysiological function. It has a highly vascularized epidermal mucosa that overlies the sphincter of Oddi. It regulates the release of bile and pancreatic enzymes and prevents reflux of enteric contents into the biliary system. Its anatomical transition is halfway along the second part of the duodenum from the foregut to the midgut, at the point where the celiac trunk stops supplying the foregut and the superior mesenteric artery takes over. We present two cases of neuroendocrine tumors (NETs) involving the ampulla and the pancreas.

## Case presentation

Case 1

**Figure 1:**
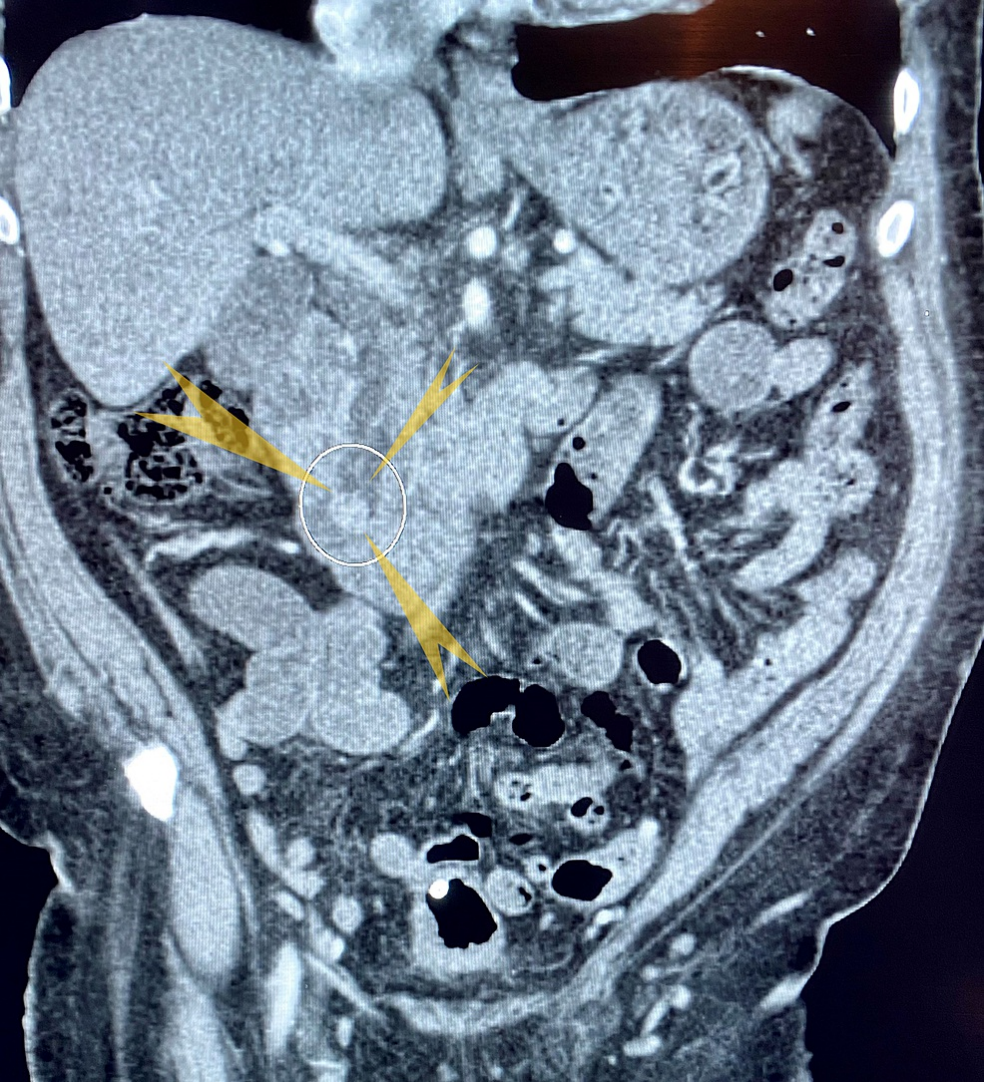
CT abdomen and pelvis with contrast showed a soft tissue density nodule seen at the level of ampulla measuring 1.2 cm X 1.1 cm (yellow arrowheads pointed towards density in the white circle) and common bile duct diameter measured 10 mm and the pancreatic duct was 4.5 mm at the level of the pancreatic head.

**Figure 2:**
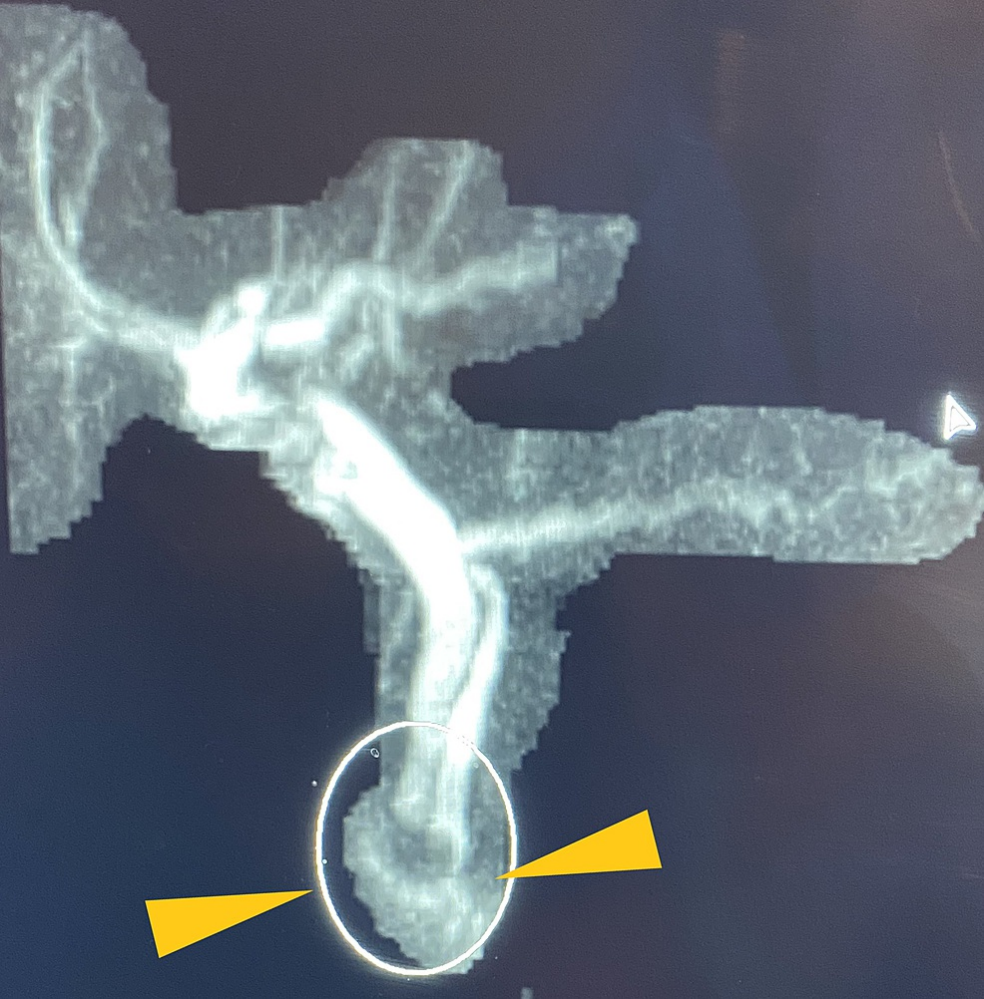
Magnetic resonance cholangiopancreatography (MRCP) showed a suspicious mass at the level of ampulla (yellow triangles).

**Figure 3:**
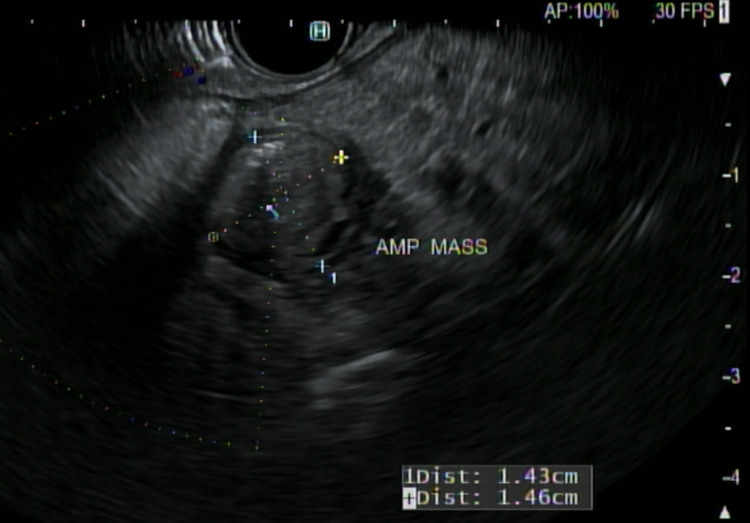
Endoscopic ultrasound shows single intramural mass in the area of papilla.

**Figure 4:**
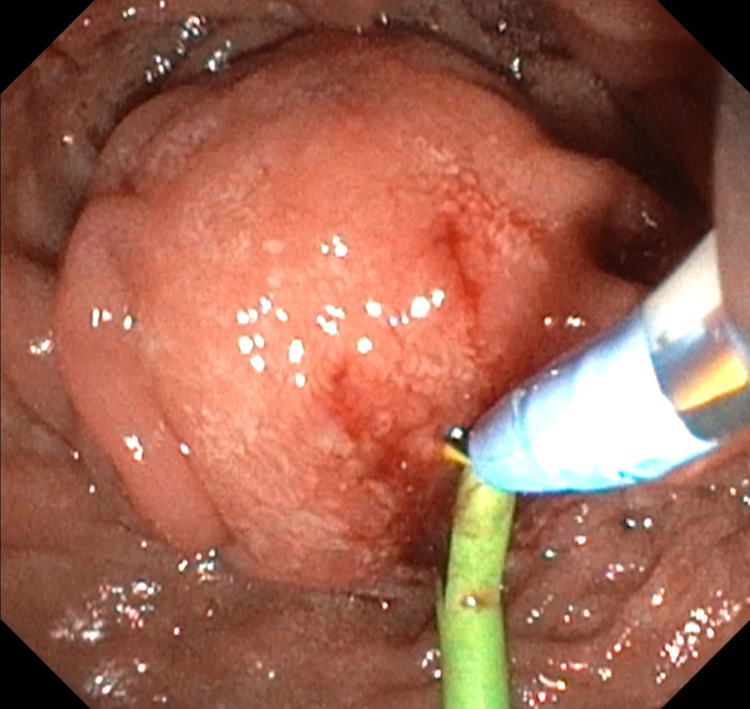
ERCP showed abnormal bulging papillae with malignant appearing partially occluding mass present in the entire biliary tract. ERCP: endoscopic retrograde cholangiopancreatography

**Figure 5:**
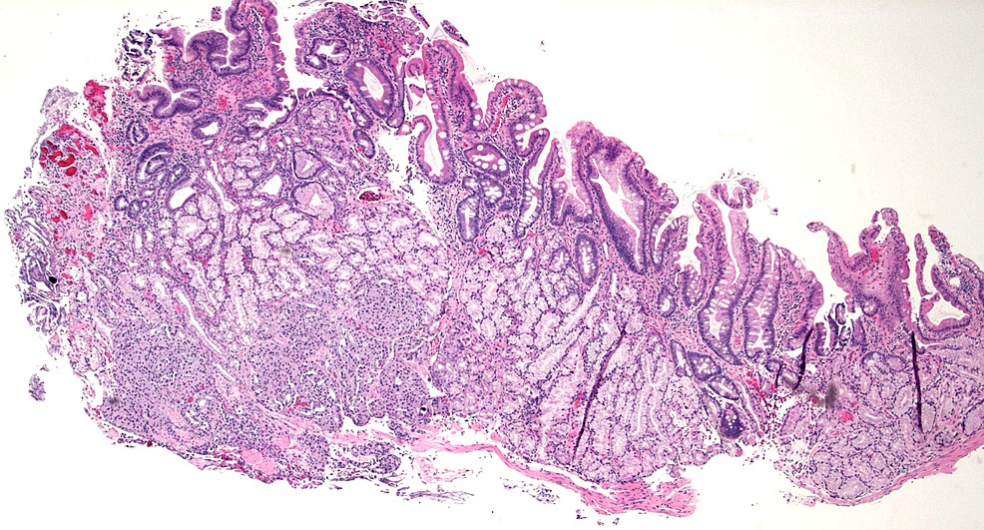
Histopathology study: Low power photomicrograph of the ampulla with nests of a well differentiated neuroendocrine tumor subjacent to Brunner's glands (Hematoxylin and Eosin stain, 40X original magnification).

**Figure 6:**
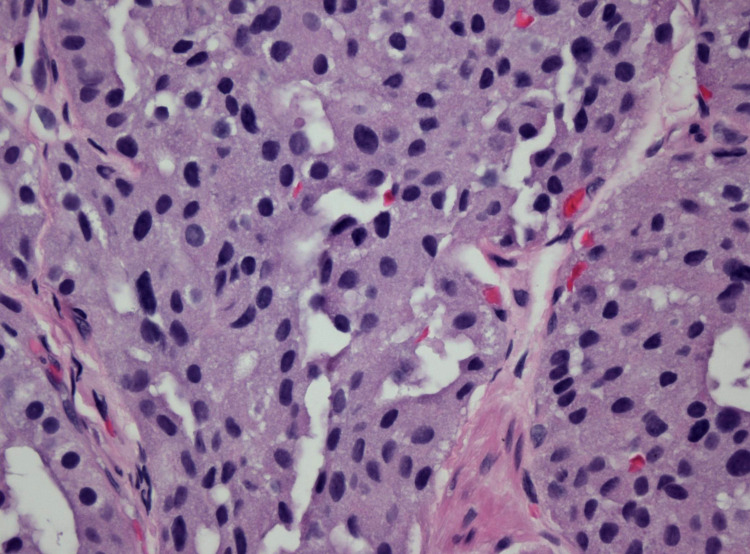
High-power photomicrograph showing the stippled chromatin and ample cytoplasm typical of a well-differentiated neuroendocrine tumor (Hematoxylin and Eosin stain, 600X original magnification).

**Figure 7:**
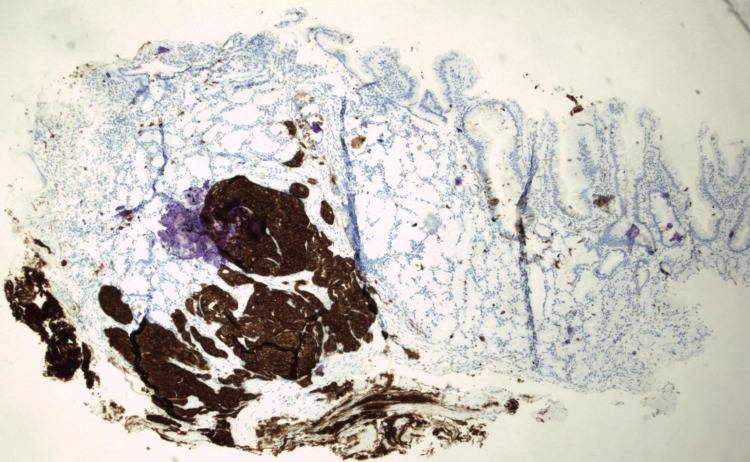
Immunohistochemical stain for synaptophysin showing strong cytoplasmic immunoreactivity within cells of the well-differentiated neuroendocrine tumor (Synaptophysin stain, 40X original magnification).

The first patient was a 71-year-old man with a significant medical history of chronic respiratory failure (requiring oxygen at home), chronic obstructive pulmonary disease, essential hypertension, a remote history of prostate cancer, and neurofibromatosis. The patient was admitted for intermittent, vague, chronic abdominal pain over the past 1 year with a 20-pound weight loss in the last 6 months. He denied loss of appetite, melena, hematochezia, and altered bowel movements. He was a known smoker but denied alcohol and illicit drug abuse. Family history was significant for breast cancer in his mother and pancreatic cancer in his father. Vitals were stable and the patient was on 5 L of supplemental home oxygen. He was anicteric, and no lymph nodes were palpable. The abdomen was soft, non-tender, non-distended and no hepatosplenomegaly or other masses were palpable. The remaining examinations were benign. Preliminary laboratory results were essentially normal, including liver function tests. Computed tomography (CT) of the abdomen and pelvis with contrast (Figure 1) showed a soft tissue density nodule at the level of the ampulla measuring 1.2 × 1.1 cm. The common bile duct diameter measured 10 mm and the pancreatic duct was 4.5 mm at the level of the pancreatic head. The gallbladder was distended with cholelithiasis. Magnetic resonance cholangiopancreatography (MRCP) showed a suspicious mass at the level of the ampulla with dilatation of the pancreatic duct measuring 5.5 mm and 3.5 mm at the level of the body (Figure 2). The patient subsequently underwent endoscopic retrograde cholangiopancreatography (ERCP) and endoscopic ultrasound (EUS; Figure 3). ERCP (Figure 4) showed abnormal, bulging papillae with a partially occluding mass with a malignant appearance. A biopsy of the mass was taken and sphincterotomy was performed, followed by stent placement. Histopathologic (Figures 5, 6, 7) studies confirmed it as a low-grade NET. The patient was scheduled for endoscopic resection. He was deemed a poor surgical candidate to undergo the Whipple procedure because of his chronic respiratory failure status.

Case 2

**Figure 8:**
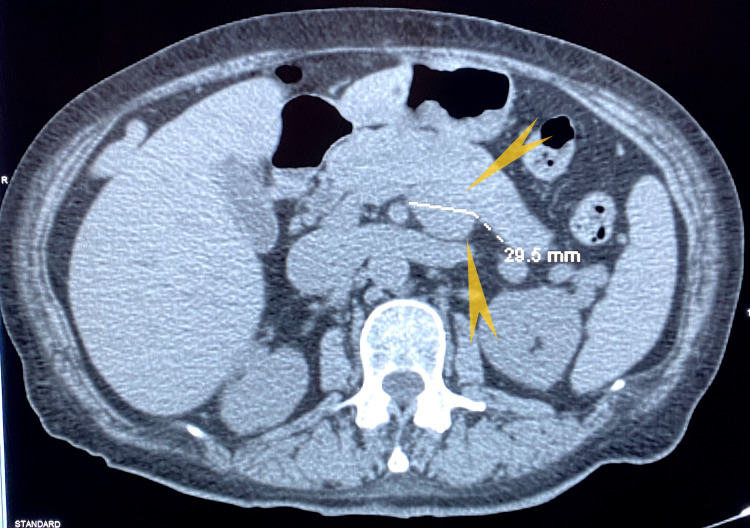
CT abdomen and pelvis w/contrast: There is a diffuse mass-like enlargement of the mid-body of the pancreas with 3 cm lobular mass projected from the superior and inferior aspects of the pancreas. Findings are highly suspicious for pancreatic neoplasm.

**Figure 9:**
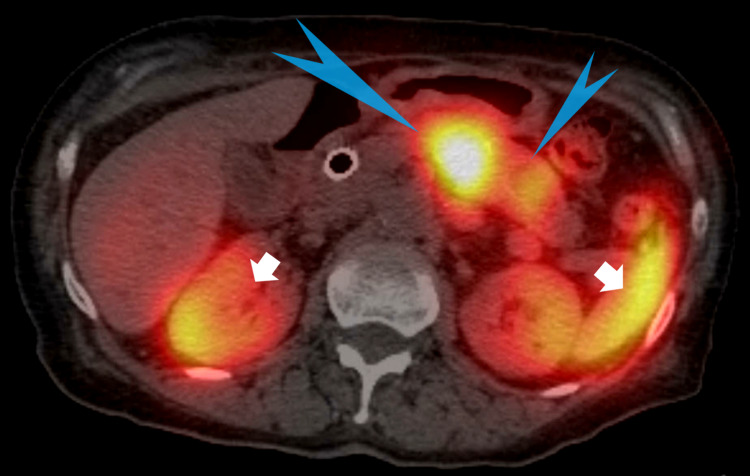
Octreoscan: Blue pointed arrowheads show marked increase uptake within the diffusely enlarged pancreas and pancreatic mass compatible with neuroendocrine tumor. Short white arrows show increased uptake in the right kidney and spleen which are normal findings in the octreoscan.

**Figure 10:**
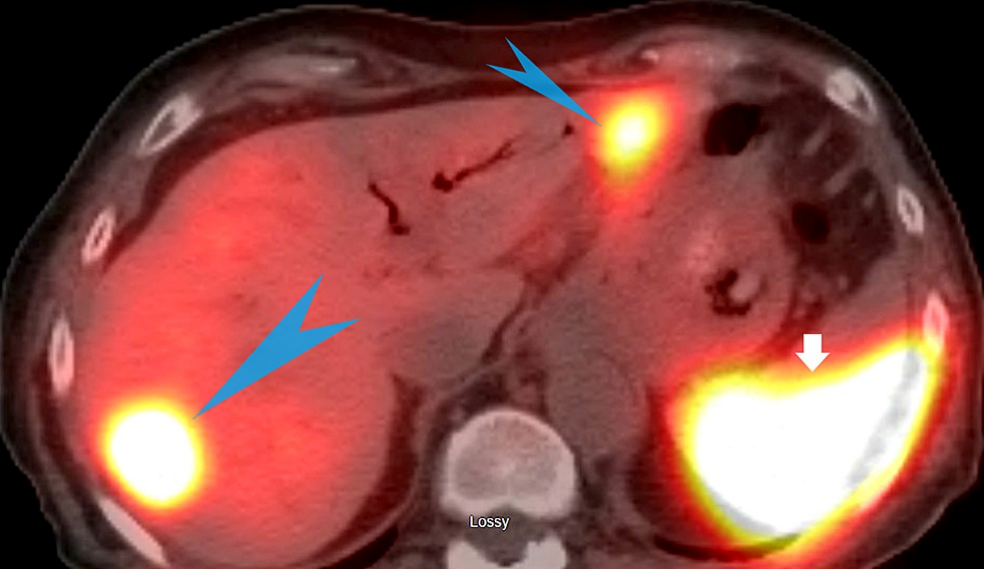
Octreoscan showing metastatic hepatic lesions: Blue arrowheads show increased uptake lesions in the lateral segment of the left lobe of the liver with another lesion in the right lobe compatible with metastasis. Short white arrow shows increased uptake in the spleen which is a normal finding.

The second patient was an 83-year-old woman with a significant medical history of essential hypertension and type 2 diabetes. The patient was admitted for intractable nausea and vomiting that had been ongoing for the preceding 4 days. The patient had been in otherwise good health before admission. She denied abdominal pain, altered bowel movements, loss of appetite, or loss of weight. She denied tobacco use and illicit drug abuse but was an occasional alcoholic. Family history was significant for; one sister had a history of breast cancer, her paternal aunt had pancreatic cancer, and another sister had a brain tumor. The patient’s vitals were stable. Her abdomen was soft and non-tender, with no hepatosplenomegaly, or other palpable masses. The remaining examinations were also benign. The laboratory results were only significant for serum alanine aminotransferase 85 U/L (reference range 16-63 U/L); aspartate aminotransferase 21 U/L (reference range 15-37 U/L); alkaline phosphatase 195 U/L (reference range 50-136 U/L); total bilirubin 0.9 mg/dL (reference range 0.2-1 mg/dL); and albumin 1.8 g/dL (reference range 3.4-5.0 mg/dL). Serum cancer antigen (CA-125) was 11 units/mL (reference range 0-35 units/mL) and cancer antigen-19 (CA-19) was 33 units/mL (reference range 0-37 units/mL). CT of the abdomen and pelvis without contrast (Figure 8) revealed a 3 cm lobular mass projecting to the superior and inferior aspects of the mid-body of the pancreas, with possible central portal vein thrombosis. MRCP showed a 17 mm diffuse dilatation of the pancreatic duct, which was filled with mixed-signal intensity soft tissue extending from the tail of the pancreas to the ampulla, arousing suspicions of a pancreatic ductal neoplasm with an associated partial obstruction of the common bile duct, which was 11 mm in diameter. The study also showed hepatic lesions suspicious for metastatic disease and probable splenic vein thrombosis. The patient underwent endoscopic ultrasound-guided fine-needle aspiration of the pancreatic mass. Histopathological studies showed small to medium cells arranged in clusters and rosettes. Cells were stained for chromogranin, synaptophysin, and cytokeratin. The immunochemistry profile favored the diagnosis of a well-differentiated, low-grade NET. Serum levels of vasoactive intestinal peptide, chromogranin, metanephrines, and glucagon levels were within normal limits. The patient underwent radiographic staging studies that included indium-111, pentetreotide (octreoscan) and gallium Ga-68 dotatate positron emission tomography (PET) scans. Octreoscan showed diffusely increased radiotracer uptake throughout the pancreatic mass (Figure 9) and three distinct areas in the liver (Figure 10), consistent with metastasis, and a single lesion in the left lung and left shoulder. Her latest dotatate PET scan showed stable lesions in the right and left hepatic lobes, a pancreatic body mass, and bone metastatic lesions. She was initially treated with everolimus and lanreotide, but everolimus had to be stopped because of weight loss. A follow-up octreoscan after 2 months showed extensive disease in the pancreas and metastasis in the liver, lungs, and bones. Subsequently, the patient was treated with temodar, which was also discontinued owing to the persistent progression of the disease. Currently, the disease is stable after the patient completed lutetium peptide receptor radioligand therapy at a tertiary oncology center.

## Discussion

NETs occur in the gastrointestinal (GI) system (73.7% of tumors), bronchopulmonary system (25.1%), and rarely in the ovaries and kidneys [3,4]. Within the GI system, the most frequent organs affected are the small bowel (28.7%), appendix (18.9%) and rectum (12.6%). NETs arise from the endocrine cells of the GI tract; these are enterochromaffin cells derived from the crypts of Lieberkühn found in the mucosa and submucosa [5].

Ampullary NETs are exceedingly rare, with an incidence <0.05% of GI NETs, in comparison with duodenal NETs, which have an incidence of 2% [6]. To our knowledge, only 150 cases of ampullary NETs have been reported in the literature [5,7]. Hatzitheoklitos et al. reported that approximately 26% of all NETs are associated with neurofibromatosis [8]. The association between NETs and this genetic disorder probably results from the transformation of an ectodermal-endodermal complex, which is also located near the ampulla of Vater [9,10]. Ampullary NETs are usually asymptomatic, and less than 3% develop into carcinoid syndrome [6,11].

Pancreatic NETs (P-NETs) are rare, constituting only 0.55% of all pancreatic tumors observed among patients aged between 40 and 60 years [12,13]. The incidence rate is 1 per 100,000 people per year. A previous study showed that the majority of patients had metastatic disease (60%), and 20% had local disease advancement at the time of diagnosis [14].

Metz et al. showed that P-NETs may occur sporadically or in association with genetic conditions such as multiple endocrine neoplasia-1 (80-100% of cases), Von-Hippel Lindau syndrome (20% of cases), and neurofibromatosis-1 (10% of cases) [15].

The current nomenclature and grading systems of NETs were proposed by the European Neuroendocrine Tumor Society in 2006-2007 and were endorsed by the World Health Organization (WHO) between 2010 and 2019 (Table 1) [16]. It is a welcome development that the nomenclature of “carcinoid” has changed to “neuroendocrine tumor” because not all NETs are benign, some are malignant and highly metastatic; in addition, not all NETs can cause carcinoid syndrome [17,18,19].

**Table 1:** Classification and grading criteria for neuroendocrine neoplasms (NENs) of the GI tract and hepatopancreatobiliary organs. LCNEC, Large‐cell neuroendocrine carcinoma; MiNEN, Mixed neuroendocrine–non‐neuroendocrine neoplasm; NEC, Neuroendocrine carcinoma; NET, Neuroendocrine tumour; SCNEC, Small‐cell neuroendocrine carcinoma, HPF: High Power Field= 2 mm3, Ki-67 index: Ki is cell proliferation index. *Mitotic rates are to be expressed as the number of mitoses/2 mm2 in areas of higher density. The Ki-67 proliferation index is based on the evaluation of ≥500 cells in areas of higher nuclear labelling (so-called hotspots). The final grade is based on whichever of the two proliferation indexes places the neoplasm in the higher‐grade category. †Poorly differentiated NECs are not formally graded, but are considered high‐grade by definition.

Terminology	Differentiation	Grade	Mitotic rate^*^ (mitoses/2 mm^2^)	Ki‐67 index^*^
NET, G1	Well differentiated	Low	<2	<3%
NET, G2		Intermediate	2–20	3–20%
NET, G3		High	>20	>20%
NEC, small‐cell type (SCNEC)	Poorly differentiated	High^†^	>20	>20%
NEC, large‐cell type (LCNEC)			>20	>20%
MiNEN	Well or poorly differentiated^‡^	Variable^‡^	Variable^‡^	Variable^‡^

Because of their rarity and their ill-defined and highly variable presentation, diagnosis of NETs is always delayed and sometimes incidental [20]. Kleinschmidt et al. and Jayant et al. reported that 60% of ampullary NETs present with jaundice because of its anatomic location, 40% as abdominal pain without jaundice; less commonly, 10% of them present with weight loss, and even less frequently, 3-6% present with acute pancreatitis. Our two patients presented with the less common presentation of abdominal pain in the ampullary NET patient, and nausea and vomiting in the P-NET patient [5,21]. 

The presentation of P-NETs depends on whether it is functional or nonfunctional. Abdominal pain (65%) is the most common presentation of non-functional NETs [22]. Additional presentations include weight loss (20-30%), anorexia and nausea (45%), and, less frequently, obstructive jaundice (17-50%) [23]. Functional P-NETs may secrete hormones such as insulin, gastrin, glucagon, vasoactive intestinal peptide, and somatostatin hormone; therefore, its presentation is dependent on the type of hormone they release.

Duodenal NETs are a differential diagnosis of tumors of the ampulla of Vater. These are categorized into five categories: duodenal gastrinoma (75%), duodenal somatostatinoma (15%), nonfunctional duodenal NETs (which comprise secretin, gastrin, or calcitonin producing tumors without a corresponding syndrome), low differentiated neuroendocrine carcinoma, and gangliocytic paraganglioma. Ampullary NET carcinoid syndrome (with symptoms of flushing, diarrhea, and asthma) is a rare presentation that occurs in only 2.8% of cases [8,9].

The sensitivity of diagnosing primary tumors is only 33% because of their size in CT and MRI studies. ERCP/EUS is the best diagnostic modality; Picus et al. reported 100% sensitivity for EUS [24]. For P-NETs, the overall sensitivity is 92% for dual-phase helical CT and 100% for EUS [25].

NET markers, such as the isolation of neuron-specific enolase, synaptophysin, and chromogranin A on histopathologic examination, are important in establishing the diagnosis. Ki-67 is a cell proliferation marker and is also a marker of tumor aggressiveness [5]. Studies have shown that Ki-67 and the proportion of proliferating cell nuclear antigen (PCNA) immunoreactivity of NETs are associated with aggressiveness and metastatic activity. These study results have identified that the higher the expression of PCNA and Ki-67, the more aggressive the biological behavior of ampullary NETs compared to duodenal NETs [26].

A review of the literature clearly recommends that Whipple’s resection with lymphadenectomy is the management of choice if the patient is suited to and can tolerate the surgery [5,27]. Another study by Baptiste et al. undertaken at Emory University in 2016 concluded that oncologic resection by pancreaticoduodenectomy is superior to trans-duodenal resection for all ampullary NETs with local lymph node involvement [28].

For patients with significant comorbidities, such as our first patient who had cardiopulmonary comorbidities (chronic respiratory failure secondary to chronic obstructive pulmonary disease), and tumor sizes <2 cm, either endoscopic resection or surgical ampullectomy is recommended and should be performed by a highly experienced surgeon because of the high associated mortality and recurrence rates [9,29,30,31].

Two studies described that the characteristics of NETs decide their aggressiveness are its size >1 cm increases the risk for lymph node involvement, but not the tumor grade, which decides the aggressiveness [32,33]. The 10-year survival rate is 71% for well-differentiated ampullary NETs, irrespective of the involvement of lymph nodes locally, but the survival rate decreases to 15% for poorly differentiated ampullary NETs [5, 34].

Prognosis is variable, and the exact prognosis of ampullary NETs is yet to be established because of the extreme rarity and the small number of reported cases. Hatzitheoklitos et al. reported an overall survival rate of 90% [8]. Two studies have shown that they have a poor prognosis, but these studies had small sample sizes [26,35].

Ampullary NETs, compared to duodenal NETs, have worse overall survival because they are more advanced at the time of presentation, but overall survival is the same for locally resected ampullary and duodenal NETs [7]. The overall survival, locally advanced disease, and metastatic disease of nonfunctional NETs at 5 years and 10 years were 47.6%, 33.7%, 60.7%, 60.7%, 32.1%, and 14.8%, respectively [14].

## Conclusions

“Carcinoids” have been renamed “neuroendocrine tumors”, according to the latest WHO guidelines. Ampullary and pancreatic NETs are exceedingly rare, their presentation is highly variable, and their diagnosis is often delayed. More data and more research are needed for the accurate assessment of prognoses; however, for all ampullary NETs irrespective of size, Whipple resection has been recommended, provided that the patient can tolerate the procedure. Endoscopic resection or surgical ampullary resection is recommended for patients who cannot tolerate it. The majority of ampullary NETs and P-NETS have metastasized by the time patients present for treatment. Therefore, we emphasize early diagnosis and management to reduce mortality and improve survival rates.
